# Impact of geochemical reactivity on desulphation requirements in a sandstone reservoir containing carbonate and sulphate minerals

**DOI:** 10.3389/fchem.2025.1540250

**Published:** 2025-01-31

**Authors:** Ali M. Al-Behadili, Eric J. Mackay

**Affiliations:** Institute of GeoEnergy Engineering, Heriot-Watt University, Edinburgh, United Kingdom

**Keywords:** desulphation, injection water composition, carbonate minerals, inorganic scale, reactive transport modelling, geochemistry, flow assurance

## Abstract

This paper presents an investigation of the impact of *in situ* chemical and geochemical interactions on oil recovery efficiency and inorganic scale management. A common technique to support the reservoir pressure is water injection, but scale problems can be a major issue that develop during oil field production when there is water (especially seawater) injection. In such flooding scenarios, geochemical reactions occur between formation and injected water in terms of sulphate scales, such as barite. On the other hand, the carbonate scales may form due to a variety of reasons: changes in temperature, pressure, pH and CO_2_ concentration in the aqueous or hydrocarbon phases. This paper investigates the impact of CO_2_ availability, and changes in pH, ionic concentrations and temperature on carbonate and sulphate scaling risk in waterflooded reservoirs where choices may be exerted over injection water composition. In this work, the injected water does not contain CO_2_, but CO_2_ is present in the oil phase, and may partition from there, or diffuse from the formation water. Also presented is the relationship between brine composition and scale precipitation and management in the production wells. There are various factors affecting the system, such as water injection well and production well flow rates and flow through the reservoir, and also compositional effects due to use of Full Sulphate Seawater (FSSW) or Low Sulphate Seawater (LSSW), and due to variations in temperature and the concentration of CO_2_ in the oil phase. In this study, as preparation for addition of geochemistry to a full field 3D history matched model, we include geochemical reactions in a 1D model that has the field pressure, temperature and fluid properties, to test the impact of the various potential reactions in a simple system. This is necessary to fully understand the system before, in future work, moving on to the full field modelling, and in fact provides very valuable learnings that would be more difficult to distil if full field modelling alone had been performed. We assume the mineral reactions (anhydrite, gypsum, barite, huntite and calcite) are in equilibrium, excepting for the magnesium rich carbonate mineral reaction, which is assumed to be kinetic. The results shows that SO_4_^2-^, Mg^2+^, HCO_3_^−^ and Ca^2+^ are the major ions that have a very significant effect on the system, and therefore impact on precipitation (4.7E-06gmole) and dissolution (-4E-06gmole) of calcite, barite and the magnesium rich carbonate mineral. Dissolution of anhydrite (−5.1E-05gmole) present in the initial mineral assemblage is shown to have a significant impact in most scenarios, except where FSSW has been heated up to reservoir temperature, where anhydrite precipitation (5E-05gmole) *in situ* occurs. This has a significant impact on the levels of desulphation that should be used to prevent sulphate scales in the production wells.

## Introduction

Rising oil prices and concerns regarding future oil supply have sparked a renewed focus on Improved Oil Recovery (IOR) and Maximising Economic Recovery (MER). One widely utilised displacement method is water flooding, and this recovery process will be key in meeting the Energy Information Agency’s (EIA) forecast world oil demand growth to 119 million barrels per day in 2025 (Hite et al., 2004).

**FIGURE 1:**
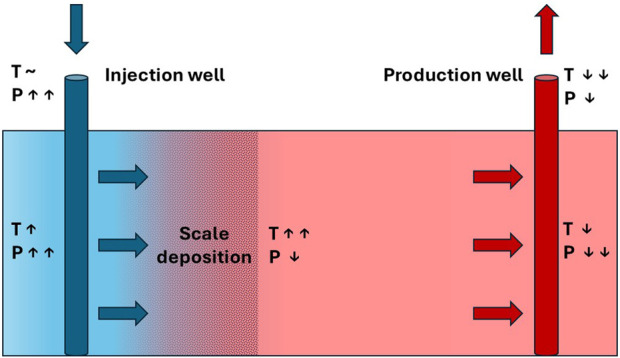
Schematic diagram of a geothermal doublet, showing the core technical installations consisting of a production and an injection well. Brine temperature (T) and pressure (P) change along the flow path. Scaling at the injection site clogs the pores, which results in reduced injectivity (after Tranter et al., 2020).

Scale problems pose significant challenges during oilfield production, especially in water-flooded fields. One of the primary issues stemming from scale deposition is the hindrance of well interventions, such as the implementation of PLT tools, and also plug setting, especially when the accessible diameter is reduced below 3–3.5”(Andersen et al., 2000). Carbonate scales are formed due to pressure decrease, and the ensuing boiling of fluid rich with carbonate causes calcite deposition on the casing wall, with a pH change that depends on the presence of CO_2_ and temperature changes (Mackay, 2003). On the other hand, sulphate scales precipitate due to the mixing between incompatible brines, such as injected and formation waters, and the ensuing effects of geochemical reactivity. Also, temperature changes may affect sulphate scale deposition and therefore loss of injectivity during produced water reinjection (PWRI), or in the injection well of a geothermal doublet, as shown in Figure 1 (Tranter, 2022; Tranter et al., 2020).

Barite (BaSO_4_) scaling is encountered in a variety of settings: it is a widespread cause of permanent formation damage in deep geothermal reservoirs. This type of scale is also among the most troublesome and expensive challenges faced in oilfield operations. Despite extensive literature on the chemical and physical properties of barite, its removal once formed remains very challenging. Many studies, e.g., (Mackay, 2002; Mackay, 2003; Jordan et al., 2008), focus on the best methods for predicting Barite scaling, such as analysing produced water and determining concentrations of Ba^2+^ and SO_4_^2-^. There are three principal factors that affect the value of the Barite solubility. These are: chemical composition of the brine, temperature and pressure (Vetter, 1975). The continuous re-injection of previously produced fluids may induce the sulphate mineral to precipitate in the host rock, as cooling reduces the solubility of Barite (Tranter et al., 2020); furthermore, produced water is sometimes topped up with seawater before re-injection during PWRI, introducing large concentrations of SO_4_^2-^.

Reactive Transport Modelling (RTM) is routinely used to predict brine compositions in production wells (Mackay, 2002; Mackay, 2003; Jordan et al., 2008). The mineral reactions to be modelled in these simulations that couple flow, and geochemical reactions are usually determined from analysis of the initial mineral assembly and from the formation and injection brine compositions. For most minerals considered the *equilibrium* constants that govern the mineral solubilities are well established and are available in the various databases that may be accessed by the reactive transport models.

However, the specified reactive surface area (RSA) of a mineral is a key factor that governs the *rate* of mineral reaction, be it precipitation or dissolution, by representing the contact surface area between the mineral and the aqueous solution per unit volume of mineral. The overall mineral reactions can be characterized by two main factors: the equilibrium constant and the rate of mineral precipitation and dissolution reactions (Jia et al., 2021). Review of the literature identifies (Landrot et al., 2012; Beckingham et al., 2016; Luo et al., 2012) two key terms, the RSA and the specific surface area (SSA), the latter being used to describe the reactivity of the pure mineral, while the RSA refers to the average reactivity of the mineral *in the given porous medium*. Therefore, the SSA value for a mineral should be converted to the RSA value as a function of the site-specific mineral volume fraction. A survey of the reactive surface area of some minerals is shown in Table 1 (Jia et al., 2021; Rauf et al., 2017; Danielik et al., 2019; Kazmierczak et al., 2022; Jones and Xiao, 2005).

**TABLE 1:** Ranges of reactive surface area (RSA) of ten minerals from literature survey.

RSA (m^2^/m^3^)	Calcite	Kaolinite	Dolomite	Quartz	Ankerite	Siderite	Illite
Low	88	17,600	560	607	521	2,008	2,528
High	6,446	2,298,400	56,146	42,313	74,030	918,585	1,238,400

RSA (m^2^/m^3^)	Gypsum	Anhydrite	Barite
Low	13,000,000	3,333	400
High	22,000,000	333,333	900

These surface areas quoted in the literature vary by two to three orders of magnitude, and in other cases not referenced here can vary by even more. These variations will depend on the rock types, but also are very sensitive to measurement type. For example, the BET method (that uses the Brunauer, Emmett and Teller theory for gas adsorption) typically requires crushing of rock samples, and results in very high surface areas, compared to history matching of reactive transport models based on observed produced reactive ion concentrations. However, as identified in (Al-Behadili and Mackay, 2024), for subsurface systems such as the ones being described in this paper, the residence times are such that calculations where reactive surface areas are 10 m^2^/m^3^ and above behave very similarly to equilibrium calculations, and thus, given the minimum reactive surface given in Table 1 is 88 m^2^/m^3^, equilibrium reactive transport modelling is used in this work.

As mentioned, combining seawater injection with produced water (PWRI) is a viable solution for offshore fields undergoing waterflooding where there is insufficient produced water to maintain adequate reservoir pressure. PWRI facilitates the disposal of produced water and supports reservoir pressure, even if it does not achieve voidage replacement. However, “topping up” with seawater also poses the risk of sulphate scaling, as produced water often contains Ba^2+^, which can mix with seawater in injection wells. To mitigate this issue, employing a Sulphate Reduction Plant (SRP) is a well-known strategy. Injectivity decline is heavily influenced by two scaling damage parameters: kinetics and formation damage coefficients (Bedrikovetsky et al., 2009). Sulphate concentrations in the brine treated by sulphate reduction plants have steadily reduced over the decades as the membrane technology has been refined. Thus, managing injected SO_4_^2-^ concentration has become a very significant tool to manage or control the sulphate scale precipitation risk. The combination of geochemical reaction modelling and reservoir simulation to accurately predict the ion concentrations at the production wells is potentially a very powerful tool that can significantly impact a scale management strategy (Jordan et al., 2008). To assess the scale risk there are two design levels: the first one relies on assessment of the scale mass and supersaturation of the brine chemistry by brine analysis and thermodynamic modelling, and secondly, to assess to what extent the risk can be managed by means of chemical or non-chemical treatments (Mackay, 2002). It is becoming increasingly important to carry out a risk analysis process for scale management as early as possible in the field development plan. Table 2 shows that one of the least soluble and hardest of the oilfield scales is barite (Mackay et al., 2004). Furthermore, calcium sulphate (CaS0_4_) scale, especially anhydrite and gypsum, still causes significant problems in many oil fields. This type of scale can cause severe plugging of equipment and producing formations; it creates the need for costly workovers and stimulation jobs. The best option to eliminate or combat this scale is often by applying scale inhibitors, for example, by inhibitor squeeze treatments before the scale is formed, which is often much more economical than repairing the damage caused by the deposition. Anhydrite deposition is less frequent, but when it does occur the mass of the deposits can be very large due to the availability of the scaling ions. One of the main sources of the ions is gypsum in the reservoir rock that may have dissolved under colder seawater injection. A second source is the presence of either or both Ca^2+^ and SO_4_^2-^ ions in the injection water (Vetter and Phillips, 1970). In general, scale deposition (carbonate or sulphate) is driven by many factors, such as changes in temperature, pH, pressure, chemical composition of formation and injection waters, and CO_2_ concentrations. The greater the availability of the above parameter values for any case, the more accurate the prediction of the scale risk will become (Tranter, 2022) (In addition to the minerals listed in Table 2, the modelling included huntite, a Mg^2+^ rich carbonate mineral, (CaMg_3_(CO_3_)_4_), which has density = 2.696 g/cc, molecular weight = 353.03 (g/mole), hardness range between (1–2) Mohs and solubility range (0.7–2.7 mg/L) (Barthelmy, 2025; Kangal et al., 2009).

**TABLE 2:** Comparison of various properties for the most common oilfield scales (after Mackay et al., 2004).

Name	Synonym	Formula	MolecularWeight	Specific gravity	Hardness (Mohs^a^)	Solubility		
						Cold water (mg/L)	Hot water (mg/L)	Other
Common scales								
barium sulphate	barite	BaSO_4_	233.39	4.5	3.3	2.22	3.36	60 mg/L in 3% HCl
calcium carbonate	calcite	CaCO_3_	100.09	2.71	3	14	18	acid soluble
strontium sulphate	celestite	SrSO_4_	183.68	3.96	3	113	140	slightly acid soluble
calcium sulphate	anhydrite	CaSO_4_	136.14	2.96	3	2,090	6,190	acid soluble
calcium sulphate	gypsum	CaSO_4_.2H_2_O	172.17	2.32	2	2,410	2,220	acid soluble
sodium chloride	halite	NaCl	58.44	2.165	2	357,000	391,200	(insoluble in HCl)
Sand grains								
silicon dioxide	quartz	SiO_2_	60.08	2.65	7	insoluble	insoluble	HF soluble

^a^ Mohs hardness scale ranges from 1 (soft, e.g., talc) to 10 (hard, e.g., diamond).

Previous studies have addressed mixing of incompatible formation and injection brines: where and when such mixing occurs evidently impacting the evolving brine composition and scale risk at the production wells (Mackay, 2002; Mackay, 2003; Mackay et al., 2004; Bedrikovetsky et al., 2009; Al-Behadili and Makcay, 2024). These are important in this study also, but emphasis is also given to brine-rock contact and brine-oil contact, since these can strongly influence the concentrations of scaling ions and the concentration of CO_2_ in the injected brine, sometimes more so than the brine-brine mixing that also takes place. Future work will use 3D modelling to consider the added impact of brine-brine mixing behaviour that can be quite complex in *heterogenous* reservoirs, but in this study 1D modelling will be used exclusively, since this is *adequate* for the purpose of characterising the brine-rock and brine-oil contact, and since 1D modelling is *necessary* for developing a *prima facie* understanding of such systems.

## Objectives

This study aims to investigate the impact of altering injection water composition on minimizing the risk of mineral scale formation at production wells in seawater-flooded reservoirs. This work accounts not only for mineral precipitation reactions *in situ* deep within the reservoir, but also dissolution reactions and the impact of CO_2_ concentration in the contacted oil phase. Reactive transport modelling is used to assess a range of scenarios and test various hypotheses about the impact of treatment options in a field case, where the initial mineralogy and the initial oil composition have a bearing on the outcomes. The impact of these parameters on the brine composition at the producer well is investigated, taking into consideration the arising risk of sulphate and carbonate scale deposition. Changes in the concentrations of scaling ions due to brine mixing and geochemical reactions deep in the reservoir as the brines approach the production well are taken into consideration. Furthermore, the effects of the following factors are considered.

• Temperature effect on the anhydrite and gypsum reactions
• Pressure and temperature impact on the huntite reaction
• Barite precipitation, and
• Calcite precipitation and dissolution

## Methodology

### Model definition

Work has been conducted on a linear 1D model of a waterflood run using the CMG GEM compositional and geochemical reservoir simulation software (CMG Ltd., 2024). The system thus modelled is synthetic, but the model is developed using data based on field in the Norwegian Sea where injection water quality is a matter of consideration. The objective is to study scale deposition for both carbonate and sulphate scaling systems, and to identify the impact that parameters such as injection water composition and temperature have on the risk of scale precipitation in production wells. The model simulates a coreflood experiment that might be carried out in a 2¼ inch long core with a 1½ inch diameter, flooded at 41.67 cc/hour (approximately 23 min to flood one pore volume). The model is run for 60 hydrocarbon pore volume throughputs, by which time all the formation water had been completely swept out, and so any ongoing reactions only occur because of disequilibrium between the injection brine and the rock. However, in none of the grid blocks in any of the simulation runs was an initially present mineral completely dissolved. (Hydrocarbon pore volume (HPV) throughput is used as an analogue for time since the injection rate is maintained constant, and since HPV throughput gives an indication of behaviour at the outlet as a function of volume of fluid injected relative to total system volume, allowing conclusions to be rescaled to the field scale).

The description of the 1D model system is summarised in Table 3 below, using metric units.

**TABLE 3:** Reservoir properties and flow controls.

Property	Values
Cartesian grid dimensionality	20 × 1 × 1 cells
Grid cell sizes (uniform)	0.00285 m × 0.033 m x 0.033 m
Bulk volume	6.51562E-05 m^3^
Porosity (homogenous)	0.25
Pore volume	1.62891E-05 m^3^
Horizontal permeability (homogenous)	500 mD
Reservoir depth (top)	3,762 m
Pressure @ 3,762 m	38,820 kPa
Temperature @ 3,762 m	136°C
Initial saturations	Swi = 0.15; Soi = 0.85; Sgi = 0
Outlet location	Cell (1,1,1)
Production liquid rate control	0.001 m^3^/day (@ reservoir conditions)
Inlet location	Cell (20,1,1)
Injection water rate control	0.001 m^3^/day (@ reservoir conditions)
Injection water temperature	18°C

The aqueous phase density is calculated using the Rowe-Chou correlation (Rowe and Chou, 1970), and the aqueous phase viscosity using the Kestin, Khalifa and Correia correlation (Kestin et al., 1981). The simulation runs for approximately 1 day of flooding. A six component Peng-Robinson Equation of State (EOS) is used. The initial composition is shown in Table 4.

**TABLE 4:** Initial oil composition.

Components	Initial global mole fraction
‘CO_2_’	0.02675000
‘CH_4_’	0.38161000
‘C2-C11’	0.41987100
‘C12-C23’	0.11723000
‘C24-C35’	0.03628900
‘C36+’	0.01825000

The water injection inlet location is cell (20,1,1) and the production outlet location is cell (1,1,1). The aqueous phase is modelled as containing 13 water soluble components (Mg^2+^, Ca^2+^, Sr^2+^, Ba^2+^, SO_4_^2-^, Na^+^, Cl^−^, Li^+^, HCO_3_^−^, CO_2_, CO_3_^2-^, OH^−^ and H^+^). Their concentrations in the formation and injection brines are presented below.

## Reactions, mineralogy and brine composition

To simulate the geochemical reactivity, both aqueous and mineral reactions are modelled.

### Aqueous reactions

Three aqueous reactions are included to model the carbonate system and capture the pH changes that will occur (Equations 1–3). These reactions are always equilibrium reactions in the model.

$${\text{OH}}^{-}+H^{+}↔H_{2}O$$ (1)

$${\text{CO}}_{3}^{2-}+H^{+}↔\;{\text{HCO}}_{3}^{-}$$ (2)

$${\text{CO}}_{2}+H_{2}O↔\;H^{+}+{\text{HCO}}_{3}^{-}$$ (3)

### Mineralogy and mineral reactions

Mineral reactions are based on the primary minerals initially present (Calcite) and any secondary reactions consistent with the ions considered at reservoir conditions (Equations 4–8).

• Initial minerals (initial volumes not significant as primary minerals never completely consumed in any grid blocks)

- Calcite:

$${Ca}^{2+}+{HCO}_{3}^{-}\;↔\;H^{+}+{CaCO}_{3}$$ (4)

occupying 10% of bulk volume.

- Anhydrite:

$${Ca}^{2+}+{SO}_{4}^{2-}↔\;{CaSO}_{4}$$ (5)

occupying 10% of bulk volume.

(precipitation possible at reservoir temperature, not at injection temperature)

• Secondary mineral reactions considered.
  - Huntite:

$${Ca}^{2+}+3{Mg}^{2+}+{4HCO}_{3}^{-}↔\;{4H}^{+}+{{CaMg}_{3}\left({CO}_{3}\right)}_{4}$$ (6)

- Gypsum:

$${Ca}^{2+}+{SO}_{4}^{2-}+2H_{2}O↔\;{CaSO}_{4}.2H_{2}O$$ (7)

(precipitation possible as temperature approaches injection temperature)

- Barite:

$${Ba}^{2+}+{SO}_{4}^{2-}↔\;{BaSO}_{4}$$ (8)

The mineral reactions are all assumed to be in equilibrium, except for the huntite reaction, which is assumed to be kinetic. The equilibrium constants are calculated as a function of temperature. The impact of the huntite reaction rate parameters has been the subject of a separate study (Al Behadili and Mackay, 2024). The Pitzer model is used to calculate component activities. Harvey’s correlation is used to calculate Henry’s constant, which is then used to calculate the solubility of CO_2_ in the aqueous phase. Henry’s constant is thus calculated as a function of pressure, temperature and salinity. This correlation supplied is applicable up to 136°C and 1,470 MPa.

### Brine compositions

Table 5 shows the compositions of formation and injection waters used in the modelling. FSSW is full sulphate seawater, and LSSW is low sulphate seawater–i.e., seawater composition after it has been treated by a Sulphate Reduction Plant. Cl^−^ is used as an inert injection water tracer, as it is not involved in any reactions.

**TABLE 5:** Water compositions used in the model.

Ions	Formation water (mg/L)	FSSW (mg/L)	LSSW (mg/L)
Na^+^	21,800	10,450	10,450
Mg^2+^	173	1,379	260
Ca^2+^	1,770	446	156
**Ba** ^**2+**^	**719**	**0**	**0**
Sr^2+^	266	2	2
Cl^−^	38,200	17,400	17,400
**SO**_**4**_^**2-**^	**0**	**3,000**	**40**
HCO_3_^−^	541	160	100

Barium and sulphate concentrations highlight, as key components in barite reaction.

CO_2_ concentration in the brine is calculated from Henry’s law and is assumed to be in equilibrium with the hydrocarbon phases, and CO_3_^2-^, OH^−^ and H^+^ are secondary ion concentrations calculated from the speciation Equations 1–3.

## Scenarios modelled

In addition to considering the differences between full sulphate and low sulphate injection water compositions (FSSW and LSSW, respectively), sensitivity to temperature was also calculated. The reservoir temperature is 136°C, but the injection temperature is only 18°C. While in full 3D reservoir simulation, non-isothermal modelling is warranted, in these 1D core scale models the assumption is made that the flow is isothermal.

The results of four scenarios are described in this paper.

• Model temperature = 18°C
  ◦ SO_4_ concentration in full sulphate seawater (FSSW) = 3,000 mg/L
  ◦ SO_4_ concentration in low sulphate seawater (LSSW) = 40 mg/L
• Model temperature = 136°C
  ◦ SO_4_ concentration in full sulphate seawater (FSSW) = 3,000 mg/L
  ◦ SO_4_ concentration in low sulphate seawater (LSSW) = 40 mg/L

## Results

**FIGURE 2:**
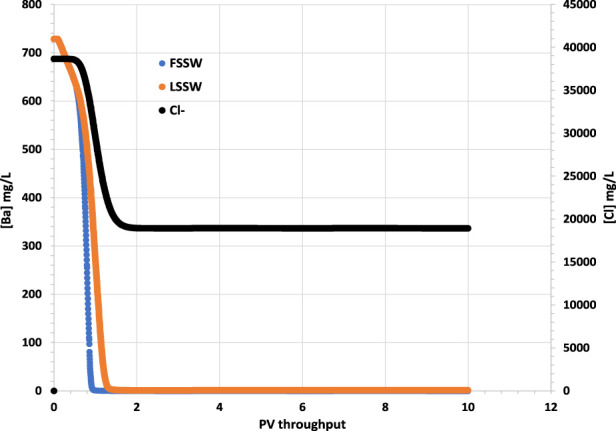
Behaviour of Ba^2+^ and Cl^−^ concentrations in the outlet block for the 18°C calculations. (in these calculations Cl-is considered to be the same in LSSW and FSSW, so that it can be used for comparing injection water breakthrough in all cases.)

The most severe scale that typically forms, in terms of difficulty to remove, is barite. The difficulty in removing it is due to its low chemical solubility and its mechanical hardness. Therefore, two important ion concentrations to monitor are those of the barium (Ba^2+^) and the sulphate (SO_4_^2-^) ions. However, in 1D modelling there is usually very little brine-brine mixing since there is only one streamline from source to sink, and therefore it is not possible to have breakthrough of SO_4_^2-^ ions on one streamline while Ba^2+^ ions are still being produced on another slower moving streamline. Figure 2 therefore shows the concentration of Ba^2+^ is initially 719 mg/L–the formation water concentration–but then decreases to near zero very rapidly (around 0.02 day) when seawater breakthrough occurs; all scenarios show the same behaviour, with a very slightly more rapid drop during FSSW injection since some of the Ba^2+^ ions are depleted by the barite precipitation reaction in the reservoir, which is greater when FSSW is injected compared to under LSSW injection.

The breakthrough of seawater can be identified from the plot of Cl^−^ in Figure 2, since Cl^*−*^ is an inert ion, and can thus be used as a tracer to differentiate seawater from formation water. In the following figures that show ion concentrations, although the model is run for 60 PV throughput, only the first 10 PV throughput is shown, since after this time ion concentrations do not vary (For plots showing mineral dissolution or precipitation, these are extended to the full 60 PV throughput since the reactions are ongoing to the end of the simulations).

It should be borne in mind that in a 3D system there will be many streamlines connecting injection and production points, and, especially in a heterogenous system, many different arrival times for injection brines, depending on each streamline; hence the Ba^2+^ concentration will decrease much less abruptly than in these 1D calculations. However, we show these to emphasize the point that for other ions it is not just brine-brine mixing that needs to be accounted for, but also brine-rock *and brine-oil interactions*.

**FIGURE 3:**
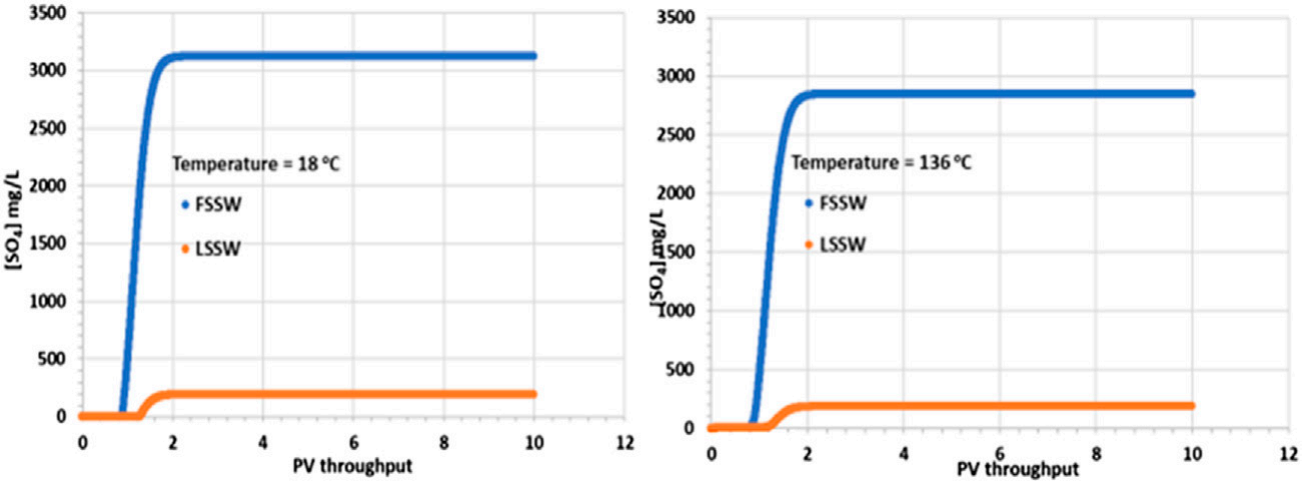
Behaviour of SO_4_^2-^ concentration in the outlet block for the 18°C (left) and 136°C (right) calculations.

Figure 3 shows that there is a corresponding increase in SO_4_^2-^ concentration on injection water breakthrough, with the increase being greater for FSSW than for LSSW, as would be expected, due to the higher concentration of SO_4_^2-^ in FSSW than in LSSW. However, closer inspection of the figure identifies that while the SO_4_^2-^ concentration in the injected FSSW is 3,000 mg/L, in the 18°C case, after breakthrough the SO_4_^2-^ concentration reaches over 3,100 mg/L, whereas in the 136°C case it reaches less than 2,900 mg/L. This indicates that in the cooler system there is dissolution of a sulphate containing mineral, while in the hotter system there is precipitation of a sulphate containing mineral.

As noted above, there may be some *in situ* precipitation of barite, but in a 1D isothermal system there is no mechanism by which barite could precipitate and then redissolve, yielding a higher concentration of SO_4_^2-^ in the outlet stream than is supplied by the inlet stream. The explanation for the SO_4_^2-^ profiles is thus that some of the primary anhydrite is dissolving into the injection brine. Since the solubility of anhydrite increases with decreasing temperature, then this process is straightforward. What is a little less straightforward is the decrease in SO_4_^2-^ concentration in the high temperature case. At higher temperature, as noted, the solubility of anhydrite is lower, generally leading to precipitation. However, there must be a source of Ca^2+^ ions for the reaction to occur, and the injection brine only has 446 mg/L of Ca^2+^. However, there is another source, which is the primary calcite mineral, and since injection brine must contact this calcite, dissolution of calcite will ensue and provide the Ca^2+^ ions required for the anhydrite precipitation reaction and the resulting drop in the produced SO_4_^2-^ concentration.

**FIGURE 4:**
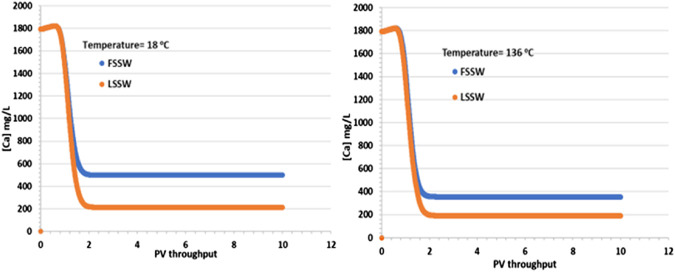
Behaviour of Ca^2+^ concentration in the outlet block for the 18°C (left) and 136°C (right) calculations.

In support of the above explanation, Figure 4 shows that there is an increase in Ca^2+^ concentrations in comparison to the injection values in both FSSW (446 mg/L) and LSSW (156 mg/L) scenarios, indicating anhydrite and/or calcite dissolution. This is true in both the higher and lower temperature models, with the effluent concentrations for the FSSW injection being greater than 500 mg/L, and for the LSSW injection being greater than 200 mg/L, an increase of approximately 50 mg/L in each case, in addition to any losses due to precipitation reactions. Furthermore, the reactions with calcite and huntite contribute to the stoichiometry, since precipitation of one mole of huntite requires four moles of CO_3_^2-^ in the solution, which in turn requires dissolution of four moles of calcite, but this leaves three additional moles of Ca^2+^ in solution, which are available to react with the injected SO_4_ to precipitate anhydrite) see Figure 4.

**FIGURE 5:**
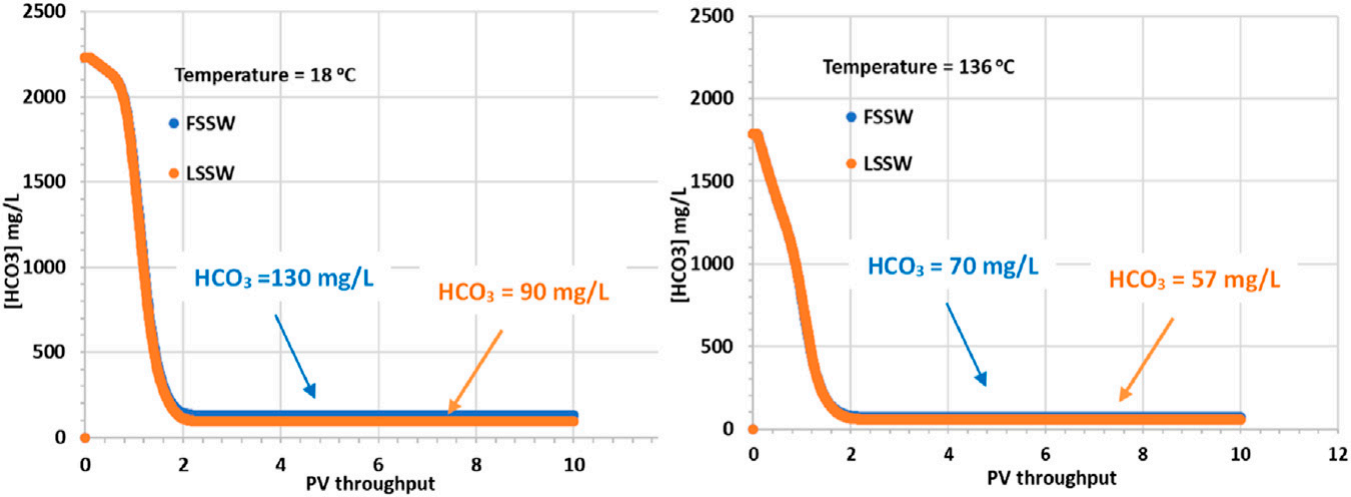
Behaviour of HCO_3_^−^ concentration in the outlet block for the 18°C (left) and 136°C (right) calculations.

In Figure 5 it is apparent that although the supplied formation water has a HCO_3_^−^ concentration of 541 mg/L, when equilibrated with the reservoir mineralogy in the presence of a CO_2_ containing oil, the equilibrated HCO_3_^−^ concentration increases, the value being very dependent on temperature. At 18°C the equilibrated HCO_3_^−^ concentration increases to over 2,200 mg/L, while at 136°C it increases to 1,800 mg/L. The HCO_3_^−^ concentrations in the injected FSSW and LSSW are much lower, 160 mg/L and 100 mg/L, respectively Table 5. Consequently, on injection water breakthrough the HCO_3_^−^ concentrations decline, but they actually decline to lower than the corresponding injection concentrations. This indicates that the balance of calcite dissolution and huntite precipitation leads to a slight decrease in HCO_3_^−^ concentrations. This is not surprising, given that, as already noted, the precipitation of one mole of huntite requires four times as many CO_3_^2-^ ions as are made available by dissolution of one mole of calcite.

**FIGURE 6:**
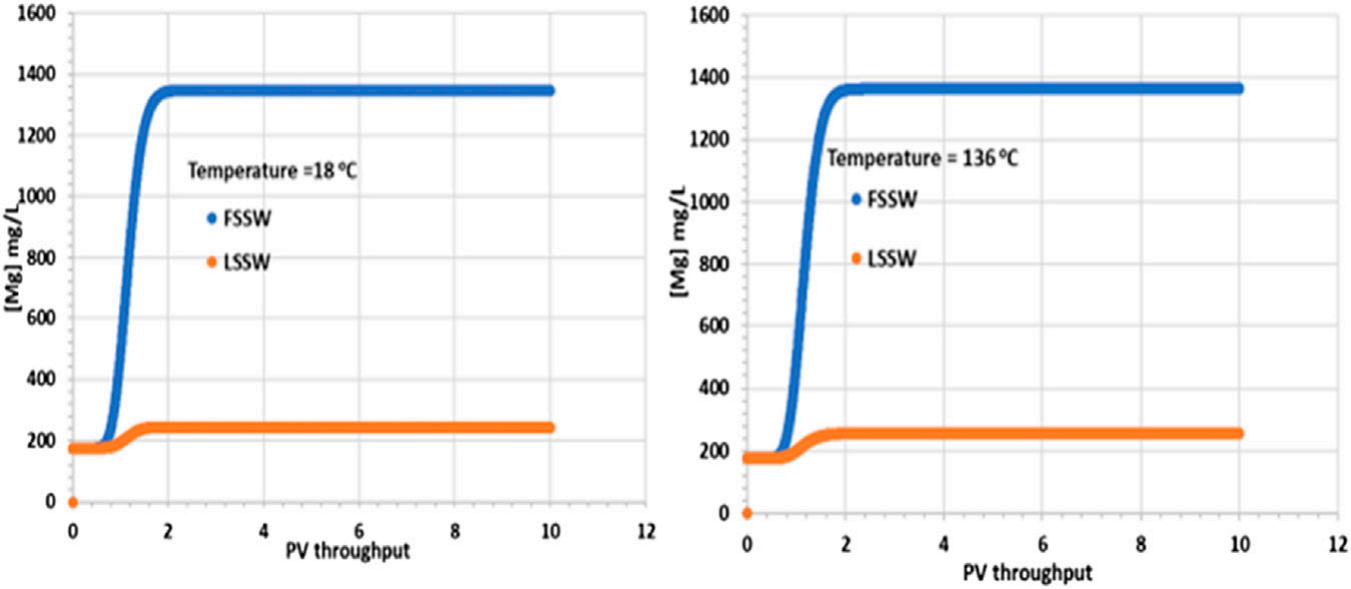
Behaviour of Mg^2+^ concentration in the outlet block for the 18°C (left) and 136°C (right) calculations.

Figure 6 shows there is an increase in the concentration of Mg^2+^ due to the injection brine having a higher concentration–be it FSSW or LSSW–than the formation brine. At reservoir temperature (which will be the dominant temperature deep within the reservoir and around the production wells) there is very little precipitation of huntite, as will be noted later. Thus, these results are consistent with the effluent Mg^2+^ concentrations closely matching the injected ones.

**FIGURE 7:**
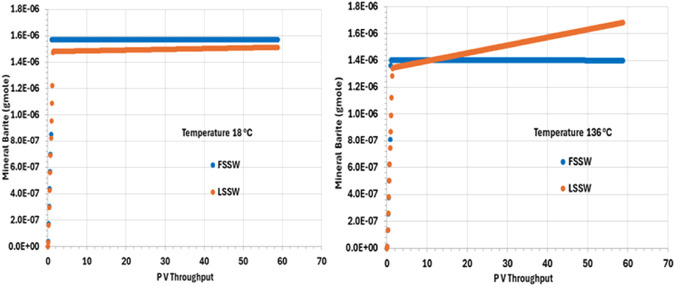
Behaviour of barite mass in the outlet block for the 18°C (left) and 136°C (right) calculations.

Figure 7 shows that there is precipitation of barite only during the very early stages of the numerical experiment, which is when the injection water interacts with the formation water. The initial deposition in the cold system is somewhat greater than in the hot system, since the solubility of barite increases as temperature increases. Indeed, perhaps somewhat unexpectedly, the amount of deposit forming is greater during LSSW injection in a hot system than it is during FSSW injection in a hot system. Although generally the reaction will be Ba^2+^ limited during FSSW injection, and SO_4_^2-^ limited during LSSW injection, in this 1D system the formation water is very quickly displaced out of each grid block as the seawater front reaches, and so FSSW and LSSW scenarios quickly become Ba^2+^ limited, and thus the impact of temperature on barite solubility becomes a more significant factor.

Additionally, during LSSW injection in the hot system there is the least amount of initial deposition, consistent with the above discussion; however, this is the only scenario where there is ongoing precipitation during the remainder of the flooding process. This is explained by there being initial precipitation of barite as brine is injected, but under these conditions, once the formation water is completely displaced, the injection is undersaturated with respect to barite, and some dissolution will take place, the greatest extent being closest to the inlet of the system. This increase in barite scaling ion concentrations around the injector will mean there is greater availability for precipitation around the producers–as noted below.

The analysis of barite precipitation needs to be undertaken cautiously, however, availability of SO_4_ is also important, and anhydrite solubility is even more temperature dependent than barite solubility.

**FIGURE 8:**
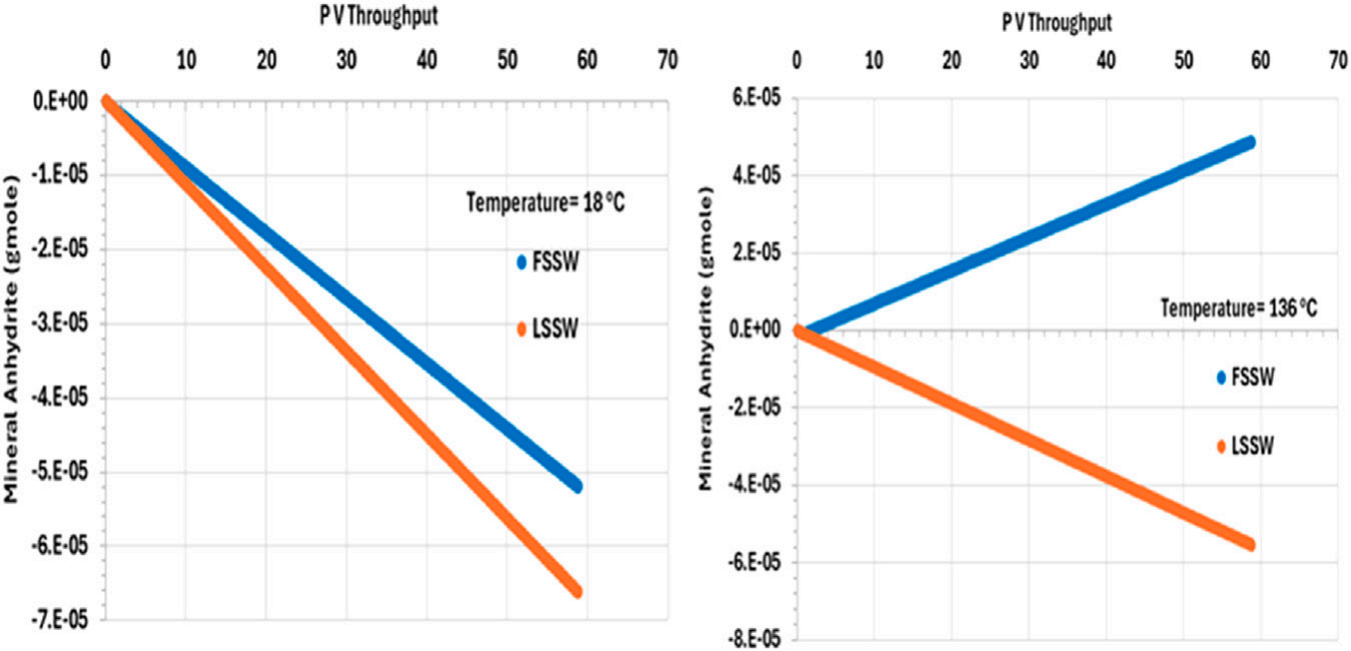
Behaviour of anhydrite mass in the outlet block for the 18°C (left) and 136°C (right) calculations.

There is an inverse relationship between temperature and anhydrite solubility–at higher temperature anhydrite is less soluble, and so lesser amounts dissolve in the injected brine. Furthermore, injection of FSSW will increase the availability of SO_4_ in solution, further reducing dissolution of anhydrite (Figure 8).

Also, Figure 8 shows the behaviour of anhydrite when the system is all at 136°C, including the injection brine. The plot shows that in the LSSW injection case, anhydrite dissolution continues to occur. However, in the case with FSSW injection at 136°C, now anhydrite precipitation takes place. This is significant, since in the reservoir thermal fronts travel more slowly that saturation fronts, and generally more slowly than compositional fronts, and so injection brine will heat up to reservoir temperature before reaching the production wells.

**FIGURE 9:**
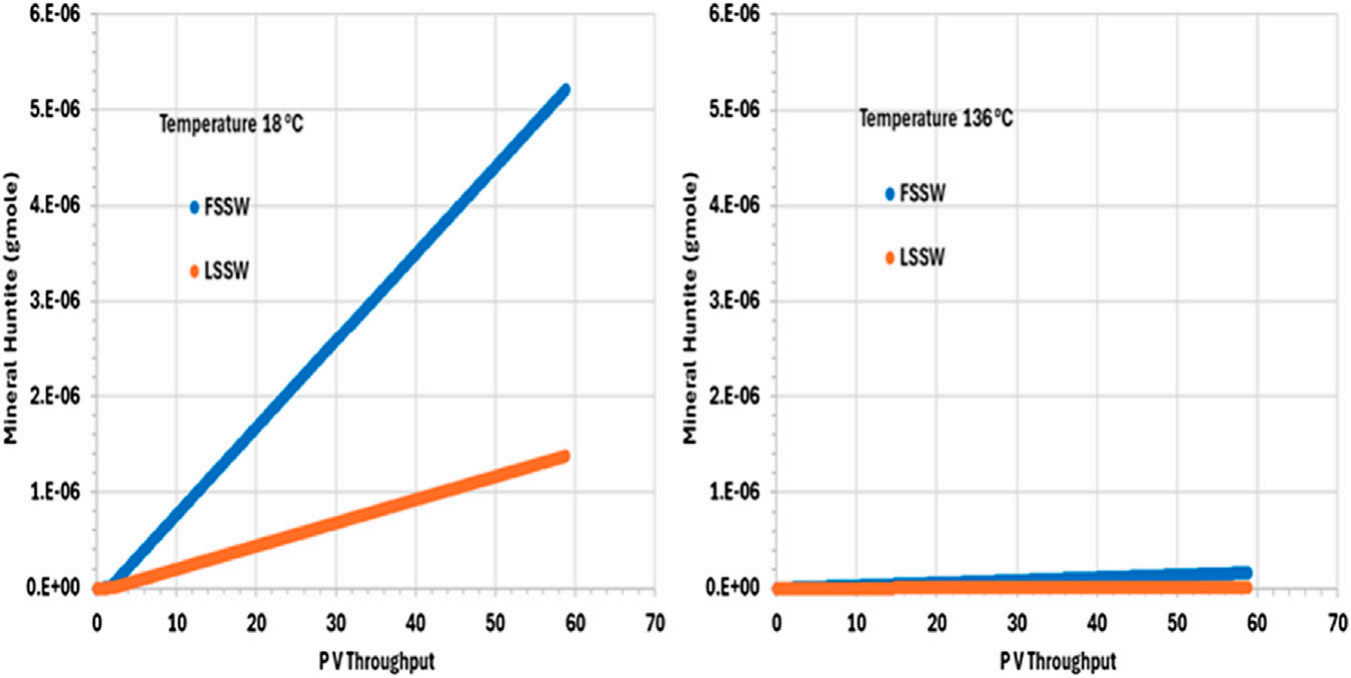
Behaviour of Huntite mass in the outlet block for the 18°C (left) and 136°C (right) calculations.

Figure 9 shows the behaviour of huntite in the outlet grid block, and from the plot we can see that in all cases huntite precipitates during waterflooding. Furthermore, the precipitation of huntite increases as temperature decreases due to the impact of temperature on the availability of Ca^2+^ (and considering that FSSW has a higher concentration of Ca^2+^ than does LSSW–see Table 5).

**FIGURE 10:**
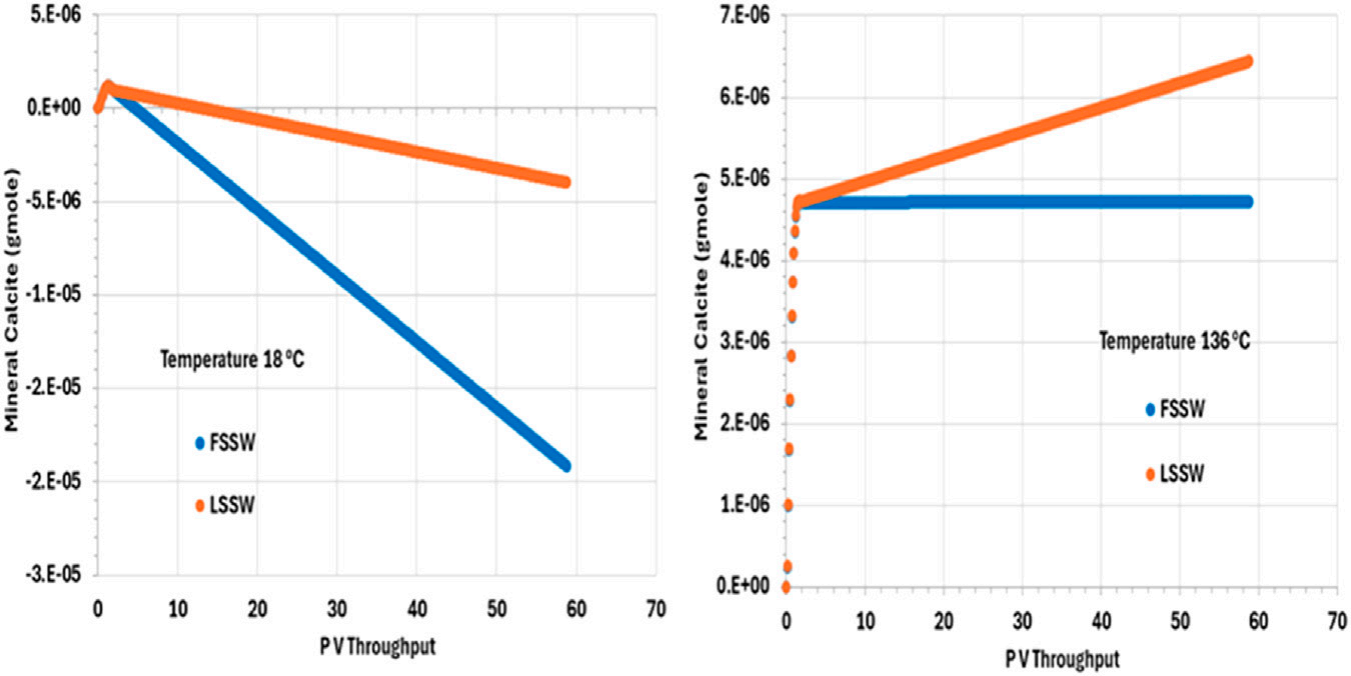
Behaviour of calcite mass in the outlet block for the 18°C (left) and 136°C (right) calculations, showing initial precipitation as pressure decreases. For high temperature injection, calcite is thereafter in equilibrium with the brine for FSSW injection, and precipitates for LSSW due to the availability of additional Ca as anhydrite dissolves. For the low temperature system, calcite dissolves to the end of the calculation, due to the increased huntite precipitation that occurs under these conditions.

Figure 10 illustrates the behaviour of calcite, showing that in all cases, after an initial short period of precipitation, calcite dissolves during waterflooding. (The short period of precipitation is attributed to the decrease in pressure in the outlet grid block as the outlet starts to flow, but this decrease in pressure is arrested once the effect of injection stabilises the overall system pressure.) The solubility of calcite is lower at higher temperatures (and decreases as CO_2_ concentration decreases–be that due to decrease in pressure or because the CO_2_ has been stripped out of the residual oil), *but*, here, more huntite precipitates at lower temperature (Figure 9), driving the calcite dissolution (since, again, precipitation of one mole of huntite requires four moles of CO_3_^2-^, which entails dissolution of four moles of calcite).

As noted, calcite solubility decreases as temperature increases. calcite dissolution in hot systems is thus less than in cold systems, everything else being equal. During LSSW injection, anhydrite continuously dissolves, leading to more Ca^2+^ being available in solution to drive greater calcite deposition, as can be seen from Figure 10.

**FIGURE 11:**
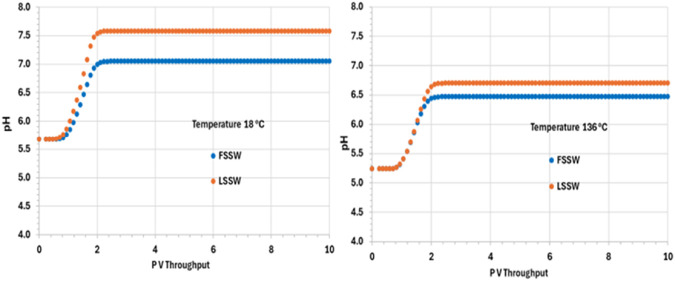
Behaviour of pH in the outlet block for the 18°C (left) and 136°C (right) calculations.

After an initial increase in pH in all the scenarios as the system equilibrates during the initial pressure change in the outlet block, the pH is thereafter buffered due to upstream interactions and remains constant at its various values in the various scenarios until the end of the calculation. In the colder system the values of pH are 7.6 and 7 in LSSW and FSSW, respectively (Figure 11). Since the aqueous and mineral reactions are strongly coupled, there are many factors that have an effect on pH: availability of CO_2_ in solution, including (declining) availability in the (residual) oil phase (see below); concentrations of Ca^2+^ and HCO_3_^−^; and the impact of any mineral reactions that impact the concentrations of these components. Whilst we may often loosely describe reactions as occurring as a consequence of there being a prevalent pH in the system, in fact the concentration of the H^+^ ion is identified by solving the series of coupled equations of which it is as much a constituent component as any other component is. From Figure 11 it is observed that pH depends on temperature, but that the choice of FSSW or LSSW injection has a greater impact on pH due to the sequence of mineral reactions that result. From Figure 8, at low temperature there is always anhydrite dissolution; at high temperature, there is anhydrite dissolution for LSSW injection, but for FSSW there is anhydrite precipitation. As noted, this impacts the availability of Ca^2+^ ions. Where there is anhydrite dissolution, then from Figure 9 we see huntite precipitation; if there is anhydrite dissolution, then there is no huntite precipitation. The huntite reaction then drives the calcite reaction (shown in Figure 10). This is straightforward at lower temperature, with more huntite precipitation driving more calcite dissolution. However, at higher temperature, for LSSW injection there is almost no huntite precipitation, and so the excess Ca^2+^ from the anhydrite dissolution causes calcite *precipitation*.

For low temperature conditions, the greater amount of huntite precipitation and calcite dissolution that occurs for FSSW injection results in a higher pH, because there are slightly more than four moles of calcite dissolved for every mole of huntite precipitated, due to the relative availability of Ca^2+^. Thus, there is some small excess of CO_3_^2-^, which drives up the pH by about 0.3 pH points (Figure 11).

For high temperatures, then for FSSW there is still a small amount of huntite precipitation (Figure 9), but little change in the calcite, so some consumption of CO_3_^2-^ which reduces the pH by 0.2 points.

**FIGURE 12:**
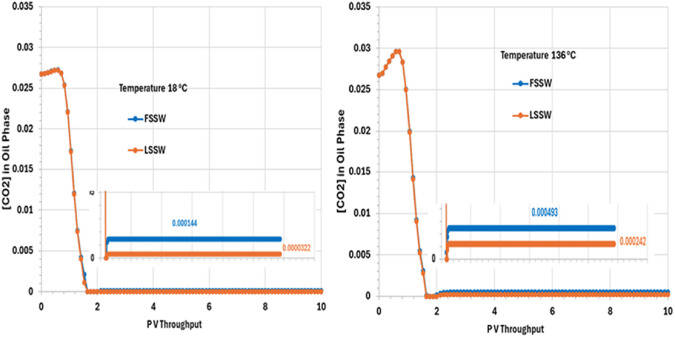
Behaviour of [CO_2_] in oil phase in the outlet block for the 18°C (left) and 136°C (right) calculations.

Figure 12 Shows that the CO_2_ concentration in oil phase decreases very quickly, within around 2 PV Throughput. This is due to partitioning of the CO_2_ from the oil into the injected water, which is assumed to have a negligible concentration of CO_2_ when it is introduced to the system. The CO_2_ concentration then remains constant to the end of the simulation, with mole fractions of around *0.000493 and 0.00242* for FSSW and LSSW, respectively, at the high temperature of 136°C, and around *0.000144 and 0.0000322* for FSSW and LSSW, respectively, at the low temperature of 18°C. This means that at higher temperatures the CO_2_ concentration in the oil phase a little bit higher than at lower temperatures; this may seem counterintuitive, but the CO_2_ concentration in the oil phase is coupled to CO_2_ concentration in the aqueous phase, and CO_2_ solubility in water is lower at higher temperatures, causing more of the CO_2_ to partition into the oil phase. There will also be the impact of any geochemical reactions with the carbonate minerals, which are also temperature sensitive. This behaviour also contributes to the explanation of the calcite behaviour (more precipitation in hot systems and more dissolution in cold systems–as per Figure 10).

In summary, in this 2D model, *in situ* mixing and temperature dependent geochemical reactions impact brine composition at the outlet, in addition to the effect of choosing to inject FSSW or LSSW, through a series of coupled aqueous and mineral reactions, as summarised in Table 6.

**TABLE 6:** Values of parameters at the outlet for each scenario (Values in *italics* are inputs, all others are calculated by the model. Initial HCO_3_ and pH values are equilibrated by the model).

Parameter	Injection	18°C		136°C		Comment
		Initial	Final	Initial	Final	
[SO_4_] mg/L	FSSW LSSW	*0* *0*	3,122 190	*0* *0*	2,900 190	(1) FSSW 136°C final [SO_4_] < injected: anhydrite precipitation. All others dissolution
[Ca] mg/L	FSSW LSSW	*1800* *1800*	500 200	*1800* *1800*	390 200	(2) Consistent with (1)
[HCO_3_] mg/L	FSSW LSSW	2,200 2,200	130 90	1800 1800	70 57	
Barite (gmole)	FSSW LSSW	*0* *0*	1.6E-06 1.5E-06	*0* *0*	1.4E-06 1.7E-06	(3) 136°C FSSW less Barite precipitation than LSSW. SO_4_ consumed by anhydrite precipitation – see (1)
Anhydrite (gmole)	FSSW LSSW	*0* *0*	−5.1E-05 -7E-05	*0* *0*	5E-05 -6E-05	(4) Confirms (1)
Huntite (gmole)	FSSW LSSW	*0* *0*	5.2E-06 1.3E-06	*0* *0*	1.6E-07 7.5E-09	(5) Huntite precipitation in all cases. More in • FSSW • 18°C
Calcite (gmole)	FSSW LSSW	*0* *0*	-4E-06 1.9E-05	*0* *0*	4.7E-06 6.5E-06	(6) Calcite precipitation in all scenarios except FSSW 18°C
pH	FSSW LSSW	5.6 5.6	7 7.6	5.2 5.2	6.4 6.7	(7) fpH dependent on temp in 136°C < 18°C
[CO_2_] in oil phase	FSSW LSSW	*0.027* *0.027*	0.000144 0.000032	*0.027* *0.027*	0.000493 0.000242	(8) More stripping of CO_2_ from oil in •LSSW •18°C

## Conclusion

### General conclusion

Core flood scale modelling has been used to understand the impact of geochemical reactions that will occur in a carbonate rich field under waterflooding in which the injection brine will have a much lower temperature than the formation, and in which choices can be made about injection water composition. In general, we note that carbonate and sulphate mineral reactions impact each other due to common ion effects, and thus a fully coupled system must be modelled: the mineral scaling reactions cannot be considered independently, neither in the 1D modelling presented here, and so, by extension, neither in full field scale 3D reactive transport modelling of the reservoir. Furthermore, CO_2_ concentration in the aqueous phase, and the impact of CO_2_ partitioning from the (mostly residual) oil phase is also important.

### Conclusion (low temperature)

When FSSW and LSSW injection brines flow through the rock at injection temperature, anhydrite dissolves. More dissolves under LSSW injection (increasing the SO_4_^2-^ concentration from 40 mg/L to ∼200 mg/L) than in FSSW (increasing the SO_4_^2-^ concentration from 3,000 mg/L to ∼3,120 mg/L). An important conclusion is thus that there is no value in desulphation to very low levels in this system, since contact with the rock will cause the SO_4_^2-^ concentration to be elevated. However, LSSW injection still brings value, as it reduces the amount of sulphate scale precipitation that may take place in the production wells.

In this setting, calcite dissolution is coupled to huntite deposition. For huntite precipitation to occur, CO_3_^2-^ ions must be made available by calcite dissolution; however, the huntite precipitation also drives calcite dissolution (the two processes are coupled and drive each other), and since the coupled reactions are CO_3_^2-^ limited, the excess of CO_3_^2-^ in huntite relative to calcite means that there is more calcite dissolution, releasing more Ca^2+^ ions to then be available for reactions involving anhydrite and/or gypsum.

Barite precipitation only occurs when the injection water mixes with the formation water in the very early phases of the numerical experiment. Because barite becomes more soluble at higher temperatures, the initial deposition in the cold system is somewhat higher than in the hot system.

### Conclusion (high temperature)

In the systems modelled at the hotter reservoir temperature, there is more calcite precipitation. Anhydrite dissolution occurs under LSSW injection, whereas under FSSW injection anhydrite now precipitates. The source of SO_4_^2-^ for the precipitation is the injected FSSW, whereas the source of the Ca^2+^ is the dissolution of the 4 mol of calcite that are required to precipitate each mole of huntite (resulting in an excess of 3 mol of Ca^2+^). As a result, the produced concentration of SO_4_^2-^ will be lower, reducing the sulphate scaling risk in the production wells. The coupled calcite and huntite reaction occur to a lesser extent under LSSW injection, indicating that the coupled anhydrite precipitation also drives the calcite dissolution (and hence huntite precipitation), since the anhydrite precipitation depletes the Ca^2+^ concentration somewhat.

Lower Ca^2+^ concentrations result in the brine being undersaturated with respect to anhydrite, leading to dissolution. Again, LSSW injection is beneficial, but there is no benefit to going to very low levels of LSSW. Identification of the optimal SO_4_^2-^ concentration will require full field 3D modelling to take account of brine-brine mixing effects as well as the brine-rock and brine-residual oil interactions investigated here.

When LSSW is injected into a heated system, more barite deposits than when FSSW is injected. In this 1D system, the formation water is rapidly displaced out of each grid block as the seawater front approaches, so even though the reaction will typically be Ba^2+^ limited during FSSW injection and SO_4_^2−^limited during LSSW injection, FSSW and LSSW scenarios quickly become Ba^2+^ limited. As a result, the influence of temperature on barite solubility becomes more significant. Furthermore, anhydrite precipitation in hot systems under FSSW flooding limits the availability of SO_4_, and this contributes to the reduction in barite precipitation in this case.

## Data Availability

The original contributions presented in the study are included in the article/supplementary material, further inquiries can be directed to the corresponding author.

## Nomenclature

### Chemical Symbols

**Ba** ^**2+**^: Barium ion
**BaSO**_**4**_: Barium Sulphate (barite)
**Ca** ^**2+**^: Calcium ion
**CaCO**_**3**_: Calcium Carbonate (calcite)
**CaMg**_**3**_ **(CO**_**3**_ **)**_**4**_: Magnesium rich Calcium Carbonate (huntite)
**CaSO**_**4**_: Calcium Sulphate (anhydrite)
**CaSO**_**4**_ **.2H**_**2**_ **O**: Calcium Sulphate (gypsum)
**Cl** ^**−**^: Chloride ion
**CO**_**2**_: Carbon Dioxide ion
**CO**_**3**_^**2-**^: Carbonate ion
**HCO**_**3**_^**−**^: Bicarbonate ion
**H** ^**+**^: Hydrogen ion
**Li** ^**+**^: Lithium ion
**Mg** ^**2+**^: Magnesium ion
**Na** ^**+**^: Sodium ion
**OH** ^**−**^: Hydroxide ion
**SO**_**4**_^**2-**^: Sulphate ion
**Sr** ^**2+**^: Strontium ion
**SrSO**_**4**_: Strontium Sulphate

### Others

1D: One dimensional (model)
3D: Three dimensional (model)
CMG: Computer Modelling Group Ltd.
FSSW: Full Sulphate Seawater
GEM: General Equation of State Model - reservoir simulation software for compositional reactive transport modelling
HPV: Throughput Hydrocarbon pore volume throughput
LSSW: Low Sulphate Seawater
PV: Pore Volume
